# Serum Uric Acid and Coronary Heart Disease in 9,458 Incident Cases and 155,084 Controls: Prospective Study and Meta-Analysis

**DOI:** 10.1371/journal.pmed.0020076

**Published:** 2005-03-29

**Authors:** Jeremy G Wheeler, Kelsey D. M Juzwishin, Gudny Eiriksdottir, Vilmundur Gudnason, John Danesh

**Affiliations:** 1Department of Public Health and Primary Care, Institute of Public HealthUniversity of CambridgeUnited Kingdom; **2**Icelandic Heart AssociationKopavegurIceland; University of SydneyAustralia

## Abstract

**Background:**

It has been suggested throughout the past fifty years that serum uric acid concentrations can help predict the future risk of coronary heart disease (CHD), but the epidemiological evidence is uncertain.

**Methods and Findings:**

We report a “nested” case-control comparison within a prospective study in Reykjavik, Iceland, using baseline values of serum uric acid in 2,456 incident CHD cases and in 3,962 age- and sex-matched controls, plus paired serum uric acid measurements taken at baseline and, on average, 12 y later in 379 participants. In addition, we conducted a meta-analysis of 15 other prospective studies in eight countries conducted in essentially general populations. Compared with individuals in the bottom third of baseline measurements of serum uric acid in the Reykjavik study, those in the top third had an age- and sex-adjusted odds ratio for CHD of 1.39 (95% confidence interval [CI], 1.23–1.58) which fell to 1.12 (CI, 0.97–1.30) after adjustment for smoking and other established risk factors. Overall, in a combined analysis of 9,458 cases and 155,084 controls in all 16 relevant prospective studies, the odds ratio was 1.13 (CI, 1.07–1.20), but it was only 1.02 (CI, 0.91–1.14) in the eight studies with more complete adjustment for possible confounders.

**Conclusions:**

Measurement of serum uric acid levels is unlikely to enhance usefully the prediction of CHD, and this factor is unlikely to be a major determinant of the disease in general populations.

## Abstract 2

The largest ever prospective analysis and meta-analysis of uric acid in coronary heart disease finds no evidence that uric acid is useful in predicting coronary heart disease

## Introduction

Numerous genetic and environmental factors have been associated with uric acid [1], and serum uric acid values are markedly elevated in patients with gout (Table 1). Since at least fifty years ago, modestly higher serum uric acid concentrations have been reported in patients with coronary heart disease (CHD) than in controls [2], and there have been suggestions that measurement of serum uric acid can enhance the prediction of CHD [3]. Prospective epidemiological studies have, however, reported apparently conflicting findings, with several studies reporting positive associations only among women [4,5], The interpretation of the data has been further complicated by the correlation of serum uric acid concentrations with several established coronary risk factors (such as blood pressure), with the use of cardiovascular medications (such as diuretics), and with clinical conditions associated with CHD (such as chronic renal disease [6]). It has been difficult, therefore, to determine whether serum uric acid values are predictive of CHD, and, if so, whether any such associations are independent from established risk factors or from the effects of disease or both.

**Table 1:**
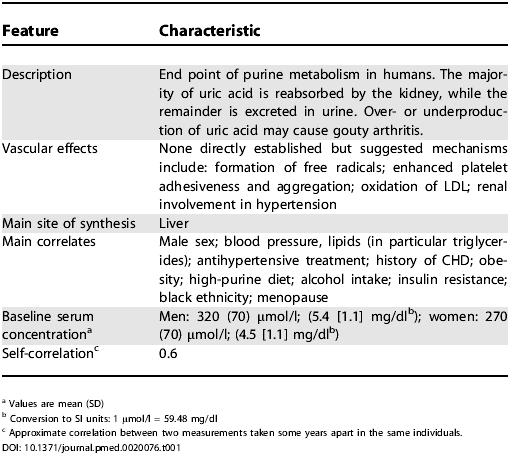
Characteristics of Uric Acid

^a^ Values are mean (SD)

^b^ Conversion to SI units: 1 μmol/l = 59.48 mg/dl

^c^ Approximate correlation between two measurements taken some years apart in the same individuals

To help address these uncertainties, we report a prospective study with more CHD cases than any previous report on serum uric acid, involving 2,459 incident cases of nonfatal myocardial infarction (MI) and CHD death, and 3,969 controls from within a prospective observational study of about 19,000 middle-aged Icelanders without a previous history of MI. To help put these results in context, we also report a meta-analysis of 15 previously published prospective studies of serum uric acid, involving a total of an additional 7,002 incident CHD cases and an additional 151,122 controls, including supplementary information obtained by correspondence from investigators to help assess in more detail the impact of possible confounders. The present analyses have been restricted to prospective cohorts sampled from essentially general populations (i.e., excluding cohorts selected on the basis of existing vascular or other diseases, or on the basis of having risk factors for vascular disease, such as high blood pressure) to reduce any distorting effects of preexisting disease on serum uric acid levels.

## Methods

### The Reykjavik Study

The Reykjavik Study, initiated in 1967 as a prospective study of cardiovascular disease, has been described in detail previously [7]. All men born between 1907 and 1934 and all women born between 1908 and 1935 who were resident in Reykjavik, Iceland, and its adjacent communities on 01 December 1966 were identified in the national population register and then invited to participate in the Reykjavik Study during five stages of recruitment between 1967 and 1991, yielding 8,888 male and 9,681 female participants without a history of MI (72% response rate). Nurses administered questionnaires, made physical measurements, recorded an electrocardiogram, performed spirometry, and collected fasting venous blood samples, which were stored at −20 °C for subsequent analysis. All participants have been monitored subsequently for all-cause mortality and for cardiovascular morbidity, with a loss to follow-up of less than 1% to date. A total of 2,459 men and women with available serum samples had major coronary events between the beginning of follow-up and 31 December 1995, yielding mean durations of follow-up among CHD cases of 17.5 (standard deviation [SD] 8.7) years and, among controls, of 20.6 (SD, 8.2) years. In total, 1,073 CHD deaths and 701 nonfatal MIs were recorded among men (including 564 confirmed MIs and 137 possible MIs), and 385 CHD deaths and 300 nonfatal MIs among women (including 237 confirmed MIs and 63 possible MIs). Deaths from coronary heart disease were ascertained from central registers on the basis of a death certificate with International Classification of Diseases codes 410–414, and the diagnosis of nonfatal MI was based on MONICA criteria. We selected 3,969 controls that were “frequency-matched” to cases on calendar year of recruitment, sex, and age in 5-y bands from among participants who had survived to the end of the study period without a MI. The National Bioethics Committee and the Data Protection Authority of Iceland approved the study protocol, and participants provided informed consent.

### Laboratory Methods

Serum uric acid levels were measured with a Technicon autoanalyzer [8]. The measurement of other biochemical analytes has been described previously [7]. Baseline measurements of serum uric acid were available on 2,456 out of 2,459 CHD cases and 3,962 out of 3,969 controls. To assess the within-person consistency of serum uric acid levels over time, measurements were made in pairs of samples collected at an interval of about 12 y apart in 379 individuals in the present study.

### Statistical Methods and Meta-Analysis

Case-control comparisons were made by unmatched stratified logistic regression fitted by unconditional maximum likelihood. Analysis of serum uric acid values was previously specified to be by sex-specific thirds of values in the controls (with subsidiary analyses involving other cut-off values). Adjustment was made for age, sex, smoking status (never, former, current), daily cigarette consumption, blood pressure, body mass index, fasting concentrations of total cholesterol and triglycerides, and various markers of socioeconomic status related to occupation, education, home ownership, and type of accommodation. We assessed variation in the strength of association according to pre-specified sub-groups, using likelihood ratio tests for interaction after adjusting for these factors, with 99% confidence intervals (CIs) used in these exploratory analyses.

For the meta-analysis, studies of serum uric acid and CHD published before May 2003 with greater than a year's follow-up conducted in essentially general populations (i.e., excluding cohorts defined on the basis of preexisting cardiovascular or other diseases) were sought by computer-based searches, scanning the reference lists of all relevant studies and review articles, hand-searching of relevant journals, and correspondence with authors of studies. Computer searches using Medline, PubMed, Web of Science, and Embase databases used keywords relating to uric acid in combination with CHD (e.g., coronary heart disease, ischemic heart disease, vascular disease, MI, and atherosclerosis). Relevant endpoints included nonfatal MI (generally using World Health Organization criteria) and CHD death (generally using International Classification of Disease criteria).

The following factors were abstracted from each study: numbers of cases and controls, mean age of cases and percentage of males, mean duration of follow-up, assay type, and those used for adjustment in multivariable assessments. Five studies were excluded because they reported insufficient data or only unadjusted risk ratios [9,10,11,12,13], but these involved only a total of about 590 CHD cases (or < 6% of the total number of cases in the present report). Of 16 studies (including four studies that had not previously reported in relation to CHD [14,15,16,17]), 11 provided supplementary tabular data on sex-specific “relative risks” (i.e., incidence rate ratios according to sex-specific thirds of serum uric acid distribution in controls) and details of factors adjusted for in multivariable analyses. We excluded female-specific estimates based on fewer than 30 CHD cases, owing to very small sample sizes from two studies [16,18]. Where data were not available by thirds of serum uric acid levels, the log-relative risk (and its standard error) was estimated from the reported relative risks using log-linear scaling and assuming normality of the uric acid distribution, as described previously [19]. Where data were available only in separate age strata, a single pooled estimate was used. Fixed-effect summary measures were calculated as the inverse-variance weighted average of the log-relative risks. Heterogeneity was assessed by the heterogeneity Q statistic and by random effect regression models with restricted maximum likelihood estimation. Subsidiary analyses (conducted only on studies known to exclude individuals with existing CHD) grouped studies by sex, study size, geographical location, sampling framework (population- or workforce-based), degree of adjustment for other cardiovascular risk factors, type of assay, and duration of follow-up. Statistical analyses were conducted using Stata version 7.0. To make some allowance for multiple comparisons, 99% CI were used for individual studies, and 95% CI were reserved for the combined estimates.

## Results

### The Reykjavik Study

The mean age at CHD event among cases was 70.2 (SD, 9.7) y. There were highly significant differences between cases and controls with respect to established vascular risk factors such as smoking, body mass index, blood pressure, and serum lipid concentrations (Table 2). Serum uric acid values were highly significantly associated with male sex, nonmanual occupation, body mass index, diastolic blood pressure, triglycerides, and serum creatinine (*p* < 0.0001 for each), although most of these associations weakened after adjustment for other vascular risk factors (Table S1). In 379 participants who provided paired blood samples, on average about 12 y apart, the within-individual correlation coefficient among serum uric acid values was 0.60 (CI, 0.54–0.66), similar to the decade-to-decade consistency observed in values of systolic blood pressure [0.66 (CI, 0.60–0.72)] and total serum cholesterol [0.60 (CI, 0.54–0.66)] in these participants.

**Table 2:**
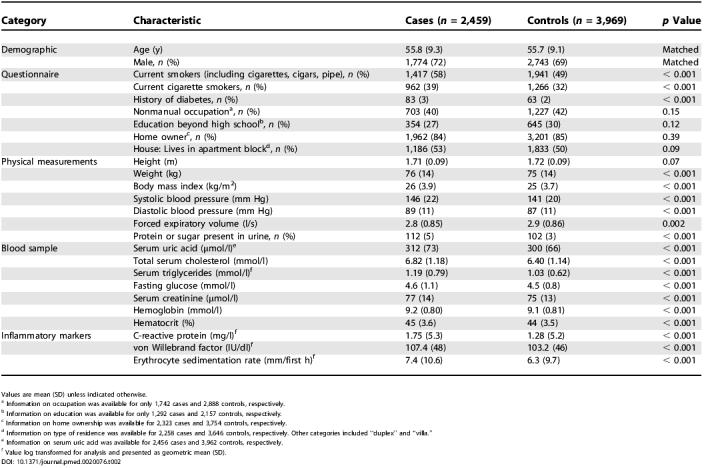
Baseline Characteristics of Cases and Controls in the Reykjavik Study

Values are mean (SD) unless indicated otherwise

^a^ Information on occupation was available for only 1,742 cases and 2,888 controls, respectively

^b^ Information on education was available for only 1,292 cases and 2,157 controls, respectively

^c^ Information on home ownership was available for 2,323 cases and 3,754 controls, respectively

^d^ Information on type of residence was available for 2,258 cases and 3,646 controls, respectively. Other categories included “duplex” and “villa.”

^e^ Information on serum uric acid was available for 2,456 cases and 3,962 controls, respectively

^f^ Value log transformed for analysis and presented as geometric mean (SD)

**Figure 1:**
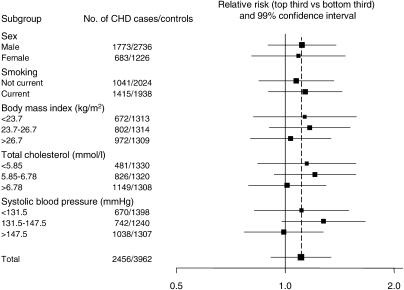
Associations between Serum Uric Acid and CHD in 2,456 cases and 3,962 Controls in the Reykjavik Study at Different Levels of Established Risk Factors Squares indicate odds ratios, with the size of the square proportional to the effective sample size.

The odds ratio for CHD was 1.39 (CI, 1.20–1.61; Wald test statistic, χ^2^_1_ = 18.4) in males in the top third compared with those in the bottom third of baseline serum uric acid levels (tertile cut-offs, > 339 versus < 286 μmol/l [Table 3]), and this fell to 1.12 (CI, 0.94–1.33; χ^2^_1_ = 1.5) after adjustment for smoking, other established risk factors, and indicators of socioeconomic status (Table 3). The odds ratio for CHD was 1.42 (CI, 1.13–1.79; χ^2^_1_ = 9.1) in females in the top third compared with those in the bottom third of baseline serum uric acid levels (tertile cut-offs, > 280 v < 232 μmol/l), and this fell to 1.12 (CI, 0.85–1.46; χ^2^_1_ = 0.6) after adjustment for smoking, other established risk factors, and indicators of socioeconomic status. In a combined analysis of males and females, the odds ratio for CHD was 1.39 (CI, 1.23–1.58; χ^2^_1_ = 27.2) and this fell to 1.12 (CI, 0.97–1.30; χ^2^_1_ = 2.4) after adjustment. In analyses restricted to the 2,083 cases without evidence of CHD at baseline, the adjusted odds ratios fell further to 1.08 (CI, 0.90–1.31) in males and 1.00 (CI, 0.75–1.33) in females (Table 3), but the findings were materially unchanged in analyses excluding the 200 CHD cases with “possible” MI or in analyses varying cut-off levels (e.g., by quarters, fifths, or increases of 1 SD; see Table 3 legend). Figure 1 indicates that there was no substantial variation in the strength of association between serum uric acid and CHD at different levels of established risk factors, and, in particular, there was no good evidence of interactions with sex or systolic blood pressure (sex, χ^2^_1_ = 0.03, *p* = 0.86; smoking, χ^2^_1_ = 0.28, *p* = 0.60; body mass index, χ^2^_2_ = 1.13, *p* = 0.57; total cholesterol, χ^2^_2_ = 2.42, *p* = 0.30; systolic blood pressure, χ^2^_2_ = 4.63, *p* = 0.10).

**Table 3:**
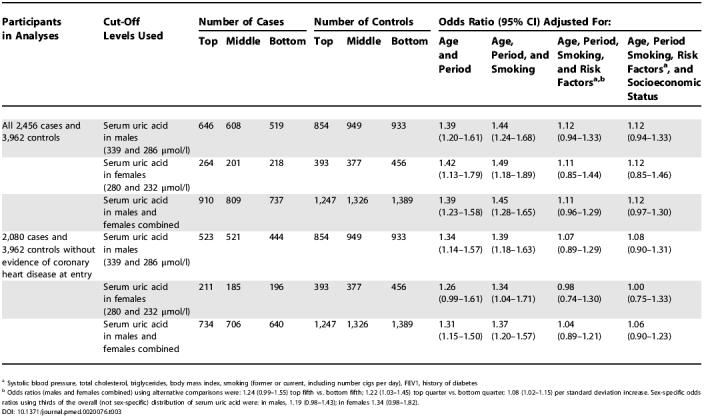
Relative Odds of Coronary Heart Disease in Individuals Who Had Serum Uric Acid in the Top Third of the Sex-Specific Distribution of Controls Relative to Those Who Had Values in the Bottom Third of This Distribution in the Reykjavik Study

^a^ Systolic blood pressure, total cholesterol, triglycerides, body mass index, smoking (former or current, including number cigs per day), FEV1, history of diabetes

^b^ Odds ratios (males and females combined) using alternative comparisons were: 1.24 (0.99–1.55) top fifth vs. bottom fifth; 1.22 (1.03–1.45) top quarter vs. bottom quarter; 1.08 (1.02–1.15) per standard deviation increase. Sex-specific odds ratios using thirds of the overall (not sex-specific) distribution of serum uric acid were: in males, 1.19 (0.98–1.43); in females 1.34 (0.98–1.82)

### Meta-Analysis

**Figure 2:**
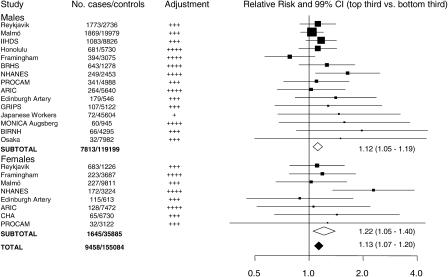
Meta-Analysis of Prospective Observational Studies of Serum Uric Acid and CHD in Essentially General Populations, Subdivided by Sex Conventions are the same as in Figure 1. Combined odds ratios and their CIs are indicated by unshaded diamonds for subtotals and shaded diamonds for grand totals. +, adjustment reported only for age and sex; ++, adjustment for these plus smoking; +++, adjustment for these plus some additional established risk factors; ++++, adjustment for these plus existing cardiovascular disease. Study abbreviations: ARIC, Atherosclerosis Risk in Communities; BIRNH, Belgium Interuniversity Research on Nutrition and Health; BRHS, British Regional Heart Study; CHA, Chicago Heart Association Detection Project in Industry; GRIPS, Göttingen Risk Incidence and Prevalence Study; IIHDS, Israeli Ischemic Heart Disease Study; MONICA, World Health Organization Monitoring Trends and Determinants in Cardiovascular Disease; NHANES, National Health and Nutrition Examination Survey; NHEFS, NHANES I Epidemiologic Follow-Up Study; PROCAM, Prospective Cardiovascular Munster Study.

**Figure 3:**
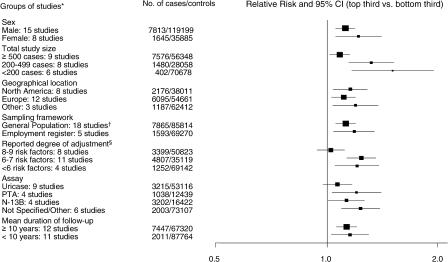
Prospective Studies of the Association of Serum Uric Acid and CHD, Grouped by Various Characteristics Conventions are the same as in Figure 1. *, each sex-specific estimate was treated as a “study”; †, two studies (6 and 13) were drawn from general practice registers; §, risk factors adjusted for included: smoking, blood pressure, total cholesterol, triglycerides, alcohol consumption, obesity, use of cardiovascular medication, history of hypertension, and history of diabetes. PTA, phosphotungstic acid.

In aggregate, 16 prospective reports [6,14,15,16,17,18,20,21,22,23,24,25,26,27,28] on serum uric acid (including the present study) have involved a total of 9,458 CHD cases and 155,084 controls, with a weighted mean age at entry of 50 y and weighted mean follow-up of 10.5 y (Table 4). Studies were conducted in the USA [22,23,24,25,28], Western Europe [6,14,15,17,18,20,27], Israel [21], and Japan [16,26], and all reported adjustment for at least smoking and some other established risk factors. Overall, in a comparison of individuals with serum uric acid values in the top third with those in the bottom third of the population, the relative risk for CHD was 1.13 (CI, 1.07–1.20: Figure 2), with statistically compatible results in male and females (χ^2^_1_ = 1.1; *p* = 0.3). In a subsidiary analysis of seven studies [20,22,23,24,25,27], involving 6,357 CHD cases and 65,978 controls, all of which excluded individuals with known cardiovascular disease at the baseline examination, the relative risk for CHD was 1.10 (CI, 1.03–1.18). There was significant heterogeneity among the 23 sex-specific study estimates (χ^2^_2_ = 38.1, *p* = 0.02), but only some of this was explained by study characteristics such as sample size (χ^2^_2_ = 11.1), geographical location (χ^2^_2_ = 1.0), sampling framework (χ^2^_1_ = 0.5), degree of adjustment for possible confounders (χ^2^_2_ = 10.0), duration of follow-up (χ^2^_1_ = 0.1), and assay type (χ^2^_3_ = 4.2) (Figure 3). In a random-effects model, that takes additional account of study variation and the joint impact of these characteristics, only degree of adjustment for possible confounders remained a significant source of heterogeneity at the 1% level of significance (sex, *p* = 0.36; sample size, *p* = 0.41; geographical location, *p* = 0.71; sampling framework, *p* = 0.02; degree of adjustment for possible confounders, *p* = 0.001; duration of follow-up, *p* = 0.67; and assay type, *p* = 0.06).

**Table 4:**
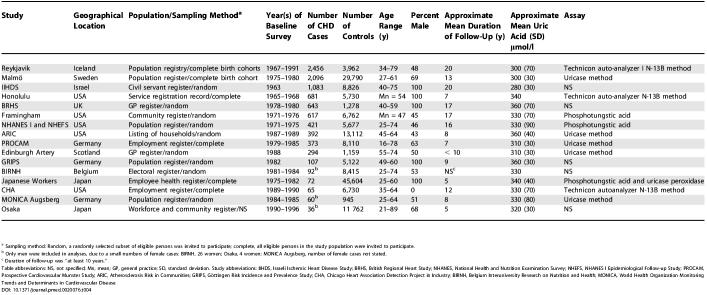
Prospective Studies of Serum Uric Acid and Coronary Heart Disease in Essentially General Populations: Study Characteristics

^a^ Sampling method: Random, a randomly selected subset of eligible persons was invited to participate; complete, all eligible persons in the study population were invited to participate

^b^ Only men were included in analyses, due to a small numbers of female cases: BIRNH, 26 women; Osaka, 4 women; MONICA Augsberg, number of female cases not stated

^c^ Duration of follow-up was “at least 10 years.”

Table abbreviations: NS, not specified; Mn, mean; GP, general practice; SD, standard deviation. Study abbreviations: IIHDS, Israeli Ischemic Heart Disease Study; BRHS, British Regional Heart Study; NHANES, National Health and Nutrition Examination Survey; NHEFS, NHANES I Epidemiological Follow-up Study; PROCAM, Prospective Cardiovascular Munster Study; ARIC, Atherosclerosis Risk in Communities; GRIPS, Göttingen Risk Incidence and Prevalence Study; CHA, Chicago Heart Association Detection Project in Industry; BIRNH, Belgium Interuniversity Research on Nutrition and Health; MONICA, World Health Organization Monitoring Trends and Determinants in Cardiovascular Disease

## Discussion

The present report provides prospective evidence from the largest study so far of serum uric acid and CHD—plus a meta-analysis of 15 previous relevant studies—involving a total of more than 9,000 incident cases and more than 150,000 controls. The overall findings suggest that individuals with baseline serum uric acid values in the top third of the population have about a 10% greater risk of CHD over the subsequent decade than those in the bottom third (with the likelihood that this association would be about twice as strong if based on long-term usual levels of serum uric acid). It is likely, however, that this modest association has been exaggerated by the preferential publication of striking findings in smaller studies (“publication bias”), or by residual confounding by established risk factors, or both. For example, the observation of weaker associations in studies with more comprehensive adjustment for possible confounders lessens the likelihood that any association between serum uric acid and CHD is independent from possible confounders; the odds ratio was only 1.02 (CI, 0.91–1.14), which is not significant, in the eight studies with the most complete reported adjustment for possible confounders (Figure 3). The present data also provide no good evidence to support previous claims that the association between serum uric acid and CHD is stronger in females than in males [5], or stronger at higher levels of established risk factors, such as in individuals with higher blood pressure recordings [29].

The main implication of these data is to refute suggestions made throughout the past half-century that measurement of serum uric acid can importantly enhance the prediction of CHD in general populations. These data do not directly address the question of whether or not serum uric acid may be involved in the causation of CHD through a number of potentially relevant vascular effects (such as through the formation of free radicals or through the oxidation of low-density-lipoprotein cholesterol [1,30]), but they suggest that serum uric acid levels are unlikely to be a major determinant of CHD.

## Supporting Information

Table S1 Comparison of Baseline Values of Risk Factors and Other Characteristics in Controls in the Reykjavik Study by Thirds of Serum Uric Acid Concentration (67 KB DOC).

**Patient Summary**

**Background**

Defining which risk factors are important for disease is useful for clinicians and patients not only because the presence of risk factors allows the prediction of who is more likely to get a disease, but also because they provide some insight into the underlying causes of disease. One such suspected risk factor for coronary heart disease is the level of uric acid in the blood. The debate over whether uric acid is useful for predicting heart disease has been going on for over fifty years. Most evidence for risk factors comes from studies of populations, in which it can be hard to tease out the effects of many different factors; often studies come to different conclusions. One way of finding out which results are reliable is to pool the results of many studies.

**What Did the Researchers Find?**

They looked at the uric acid levels of around 2,500 people with coronary heart disease and almost 4,000 controls measured at the start of a large study in Iceland, and then investigated whether there was a relation between levels of uric acid and development of heart disease. After adjusting for all the other factors that could affect the chance of heart disease, they found that uric acid did not predict heart disease. They then combined these results with those from 15 other studies, and confirmed the findings.

**What Do These Findings Mean?**

After fifty years, it now seems clear that measurement of uric acid does not help to predict heart disease. It may still be involved in triggering heart disease, but any effect must be subtle.

**Where Can I Get More Information?**

The National Heart Lung and Blood Institute has many pages of information on heart disease: http://www.nhlbi.nih.gov/health/public/heart/index.htm#ami

## Abbreviations

CHD: coronary heart disease
CI: confidence interval
MI: myocardial infarction
SD: standard deviation
