# Supplementary material for: Serum Uric Acid and Coronary Heart Disease in 9,458 Incident Cases and 155,084 Controls: Prospective Study and Meta-Analysis
Source: PLoS Med. 2005 Mar 29;2(3):e76. doi: 10.1371/journal.pmed.0020076 (PMC1069667; doi:10.1371/journal.pmed.0020076)
Supplement: Table S1 — (67 KB DOC). [file pmed.0020076.st001.doc]

**Table S1. Comparison of baseline values of risk factors and other characteristics in controls in the Reykjavik study by thirds of serum uric acid concentration**

Figures are mean (SD) unless indicated otherwise.

|  | **Top third** (>321 μmol/l) | **Middle third** (268-321μmol/l) | **Bottom third** (<268 μmol/l) | **t value^‡^** (age, sex, period adjusted) | **t value^‡^** (further adjusted^+^) |
| --- | --- | --- | --- | --- | --- |
| **Demographic** |  |  |  |  |  |
| Age (years) | 56 (9) | 55 (9) | 56 (9) | 0.9 | -1.1 |
| Male, n (%) | 1139 (87) | 953 (76) | 644 (46) | 21.6 | 15.2^***^ |
| **Questionnaire** |  |  |  |  |  |
| Current smokers (includes cigarettes, cigars, pipe), n (%) | 609 (46) | 626 (50) | 703 (50) | -4.1 | -3.5^*^ |
| Current cigarette smokers, n (%) | 355 (27) | 377 (30) | 532 (38) | -3.5 | -2.7^*^ |
| History of diabetes, n (%) | 14 (1) | 16 (1) | 33 (2) | -3.0 | -3.4^*^ |
| Non-manual occupation, n (%) | 503 (47) | 413 (42) | 308 (38) | 5.5 | 4.5^***^ |
| Education beyond high school, n (%) | 275 (34) | 207 (29) | 161 (25) | 2.1 | 1.7 |
| Home owner, n (%) | 1043 (84) | 1036 (88) | 1117 (85) | -1.1 | -1.4 |
| House:lives in apartment block, n (%) | 571 (47) | 570 (50) | 687 (53) | -1.5 | -1.2 |
| **Physical measurements** |  |  |  |  |  |
| Height (m) | 1.74 (.08) | 1.73 (.08) | 1.68 (.09) | 1.7 | 1.7 |
| Weight (kg) | 81 (13) | 76 (12) | 68 (12) | 18.2 | 12.3^***^ |
| Body mass index (kg/m^2^) | 27 (4) | 25 (3) | 24 (4) | 19.1 | 12.4^***^ |
| Systolic blood pressure (mm Hg) | 145 (20) | 141 (20) | 139 (19) | 9.6 | 3.0^*^ |
| Diastolic blood pressure (mm Hg) | 90 (11) | 87 (11) | 85 (10) | 11.5 | 4.0^***^ |
| Forced expiratory volume (l per sec) | 3.0 (0.8) | 3.0 (0.8) | 2.6 (0.8) | -1.8 | -2.5 |
| Protein or sugar present in urine, n (%) | 33 (3) | 28 (2) | 41 (3) | -1.6 | -2.2 |
| **Blood sample** |  |  |  |  |  |
| Total serum cholesterol (mmol/l) | 6.43 (1.08) | 6.38 (1.13) | 6.40 (1.22) | 6.8 | 1.7 |
| Serum triglycerides (mmol/l) ^§^ | 1.21 (0.87) | 1.03 (0.56) | 0.87 (0.49) | 17.7 | 11.6^***^ |
| Fasting glucose (mmol/l) | 4.6 (0.7) | 4.5 (0.7) | 4.4 (0.9) | 4.0 | 0.5 |
| Serum creatinine (µmol/l) | 81 (13) | 76 (11) | 69 (11) | 14.2 | 10.6^***^ |
| Haemoglobin (mmol/l) | 9.3 (0.7) | 9.1 (0.8) | 8.7 (0.8) | 8.3 | 3.1^*^ |
| Haematocrit (%) | 45 (3) | 45 (3) | 43 (4) | 7.4 | 3.0^*^ |
| ESR (mm/1^st^ h) ^§^ | 6.1 (10) | 5.8 (10) | 7.0 (10) | 6.3 | 3.9^***^ |
| C-reactive protein (mg/l) ^§^ | 1.60 (6.1) | 1.23 (4.2) | 1.08 (5.0) | 8.9 | 3.9^***^ |
| Von Willebrand factor (IU/dl) ^§^ | 106.1 (48) | 102.7 (46) | 101.0 (45) | 2.6 | 1.7 |

^+^ adjusted for age, sex, period of recruitment, systolic blood pressure, total cholesterol, triglycerides, body mass index, FEV1,

smoking (never, former or current, and number cigarettes per day), except analyses of height and weight were not adjusted for BMI, and analysis of diastolic blood pressure was not adjusted for systolic blood pressure.

^§^ factor log transformed for analysis and presented as geometric mean (SD)

^‡^ test for trend in levels of the risk factor over the groups defined by serum uric acid thirds

^*^ p<0.01

^**^ p<0.001

^***^ p<0.0001
